# Design, Synthesis, and Antimicrobial Evaluation of Novel Pyrazoles and Pyrazolyl 1,3,4-Thiadiazine Derivatives

**DOI:** 10.3390/molecules23092092

**Published:** 2018-08-21

**Authors:** Ibrahim Ali M. Radini

**Affiliations:** Chemistry Department, Faculty of Science, Jazan University, Jazan 2097, Saudi Arabia; iradini44@gmail.com; Tel.: +966-566-644-4196

**Keywords:** pyrazole-1-carbothiohydrazide, 1,3,4-thiadiazines, hydrazonyl chlorides, antimicrobial activity, MIC

## Abstract

A novel series of pyrazolyl 1,3,4-thiadiazines **5a**–**c**, **8a**–**c**, **12**, **15a**–**c**, **17a**–**c**, and **20** was prepared from the reaction of pyrazole-1-carbothiohydrazide **1a**,**b** with 2-oxo-*N*′-arylpropanehydrazonoyl chloride, 2-chloro-2-(2-arylhydrazono)acetate, and 3-bromoacetylcoumarin. Moreover, the regioselective reaction of 5-pyrazolone-1-carbothiohydrazide **1a** with 4-substituted diazonium salts and 4-(dimethylamino)benzaldehyde gave the corresponding hydrazones **21a**–**c** and **22**. The newly prepared compounds were characterized by spectroscopy and elemental analysis. Many new synthesized compounds showed considerable antimicrobial activity against tested microorganisms. Hydrazones **21a**–**c** and **22** showed remarkable antibacterial and antifungal activities. 4-(2-(*p*-tolyl)hydrazineylidene)-pyrazole-1-carbothiohydrazide **21a** displayed the highest antibacterial and antifungal activities with minimum inhibitory concentration (MIC) values lower than standard drugs chloramphenicol and clotrimazole, in the range of 62.5–125 and 2.9–7.8 µg/mL, respectively.

## 1. Introduction

**Figure 1:**
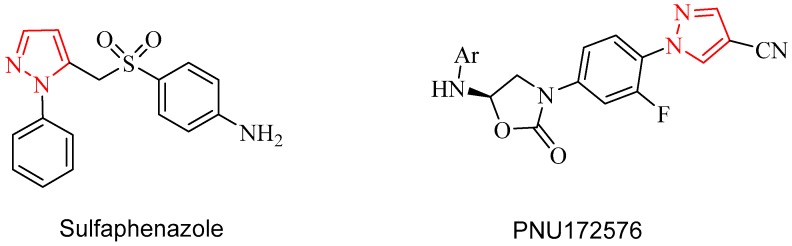
Representative structures having pyrazole moieties as antimicrobial agents.

Recently, the incidence of microbial infections had been increased dramatically because of the misuse of antibiotics has caused the pathogens to become resistant to them and which has led to serious health hazards [1]. The rate of bacterial resistance to antibiotics is higher than the rate of development of new classes of antibiotics [2] so the design and synthesis of new compounds have potential antimicrobial activity are very important issue. Pyrazole derivatives have great attention due to their interesting biological and pharmaceutical activities such as antidepressant [3], antioxidant [4], anti-inflammatory [5], anticancer [6], antimicrobial [7,8,9], antiviral [10,11], anticonvulsant [12], and insecticidal activities [13]. In addition, the natural pyrazole C-glycoside, pyrazofurin (4-hydroxy-3β-d-dribofuranosyl-1*H*-pyrazole-5-carboxamide) has a broad spectrum of antimicrobial, antiviral, and antitumor activities [14]. It is well known that pyrazoles possess significant antibacterial activity. There are many antibiotic drugs containing pyrazole moiety such as Sulfaphenazole and PNU172576 (Figure 1).

Heterocycles containing the thiadiazines moiety have biological and pharmaceutical importance [15,16,17,18]. Recently, Khidre et al. [19] reported that 1,3,4-thiadiazine derivatives have a good antimicrobial activity. Motivated by the preceding information and continuation of my research program on the synthesis of novel bioactive heterocycles [7,20,21,22,23] I designed and synthesized a novel series of pyrazole and pyrazolyl 1,3,4-thiadiazine derivatives, for antimicrobial evaluation, starting from pyrazole-1-carbothiohydrazide **1a**,**b**.

## 2. Results

### 2.1. Chemistry

**Scheme 1:**
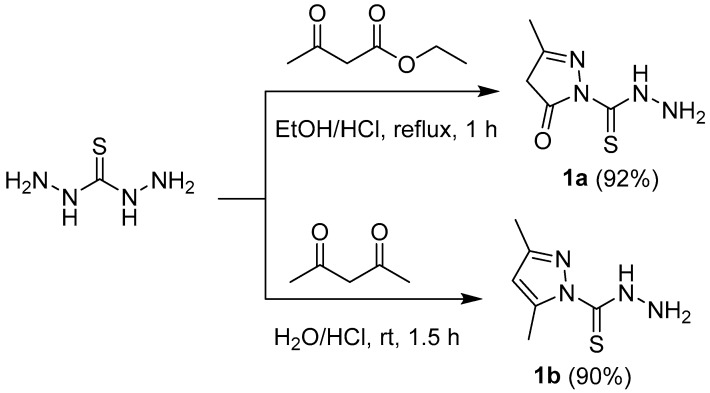
Synthesis of pyrazole-1-carbothiohydrazides **1a** and **1b**.

**Scheme 2:**
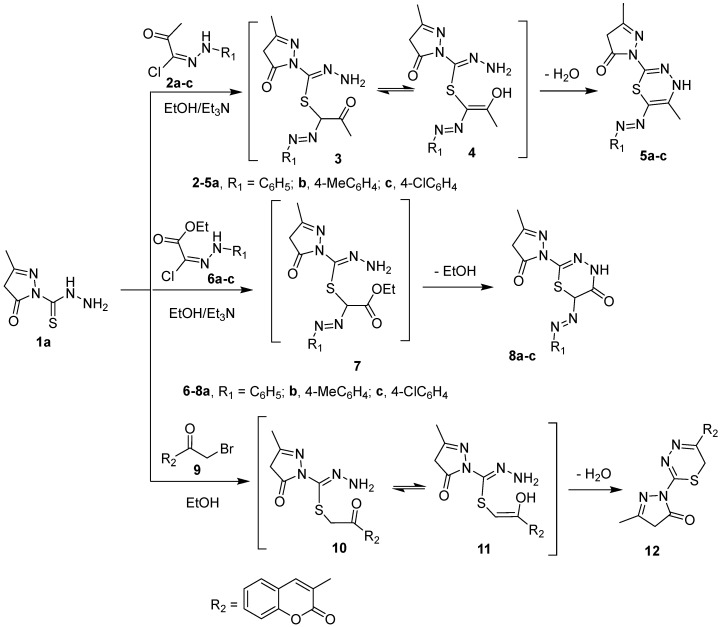
Synthesis of compounds **5a**–**c**, **8a**–**c**, and **12**.

**Scheme 3:**
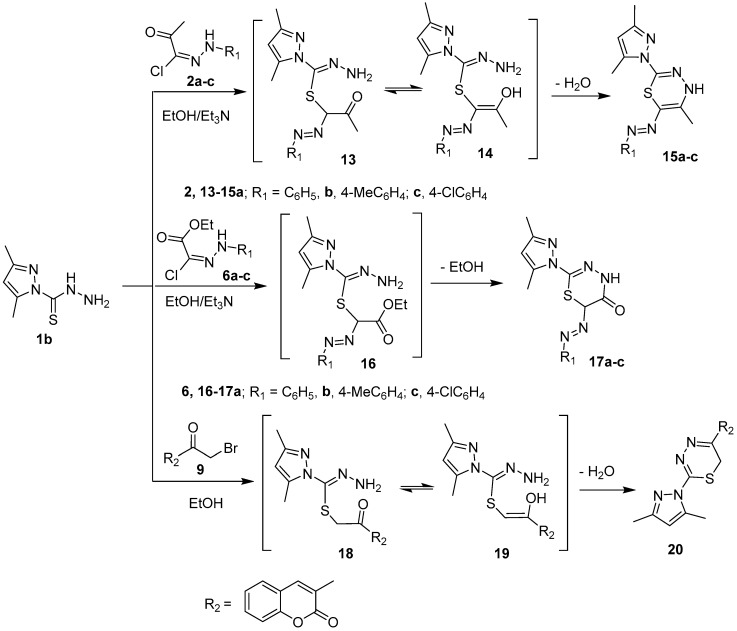
Synthesis of compounds **15a**–**c**, **17a**–**c** and **20**.

**Scheme 4:**
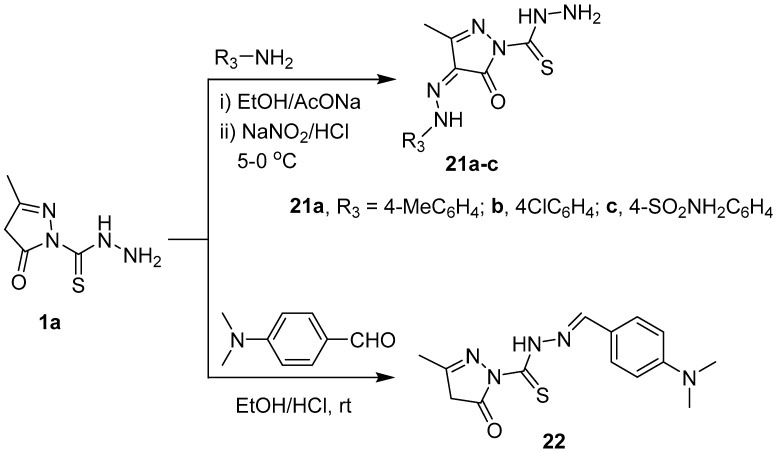
Synthesis of compounds **21a**–**c** and **22**.

My strategy to synthesize a new heterocyclic compounds involved the use of pyrazole-1-carbothiohydrazide **1a**,**b** that contains a number of chemically distinct functionalities, which can be reacted with different hydrazonyl chlorides, α-haloketones, diazonium salts, and aldehydes to a library of molecular diverse compounds (Scheme 1, Scheme 2, Scheme 3 and Scheme 4). As illustrated in Scheme 1, 3-methyl-5-oxo-4,5-dihydro-1*H*-pyrazole-1-carbothiohydrazide **1a** was prepared in a very good yield by reaction of equimolar amount of ethyl acetoacetate with thiocarbohydrazide in ethanol containing a catalytic amount of HCl at reflux temperature. Treatment with a mixture of thiocarbohydrazide in (HCl, 0.05 M) with acetyl acetone at RT for 1.5 h afforded 3,5-dimethyl-1*H*-pyrazole-1-carbothiohydrazide **1b [24]**. The molecular structure of compounds **1a** was confirmed by elemental analyses and spectroscopic methods. The infrared spectrum of **1a** showed a characteristic bands at 3292, 3250, 3182, and 1685 cm^−1^ due to NH, NH_2_, and C=O functions, respectively. ^1^H-NMR revealed two singlet peaks at 2.02 and 3.28 ppm due to the CH_3_ and CH_2_, respectively. Also, molecular weight determination (MS) of **1a** showed the molecular ion peaks at *m*/*z* 172.

The carbothiohydrazide moiety in **1a**,**b** was reacted with selected electrophiles to prepare pyrazolyl 1,3,4-thiadiazine derivatives. Pyrazole-1 carbothiohydrazide **1a** was reacted with 2-oxo-*N*′-arylpropanehydrazonoyl chlorides **2a**–**c** in hot ethanol in the presence of Et_3_N to yield 5-methyl-2-(5-methyl-6-(aryldiazenyl)-4*H*-1,3,4-thiadiazin-2-yl)-2,4-dihydro-3*H*-pyrazol-3-ones **5a**–**c**, in good yields, via intermediates **3** and **4** (Scheme 2). Similarly, 2-(3-methyl-5-oxo-4,5-dihydro-1*H*-pyrazol-1-yl)-6-(phenyldiazenyl)-4*H*-1,3,4-thiadiazin-5(6*H*)-ones **8a**–**c** were synthesized, in high yields, from the reaction of **1a** with ethyl 2-chloro-2-(2-arylhydrazono)acetate derivatives **6a**–**c** under the same reaction conditions described for the preparation of **5a**–**c**. 5-methyl-2-(5-(2-oxo-2*H*-chromen-3-yl)-4*H*-1,3,4-thiadiazin-2-yl)-2,4-dihydro-3*H*-pyrazol-3-one **12** is furnished in a good yield when **1a** was refluxed with 3-bromoacetylcoumarin **9** in ethanol (Scheme 2).

The structure of the compounds **5**, **8**, and **12** was confirmed by elemental analyses and spectroscopic methods. The IR spectrum of thiadiazinyl pyrazolone **5c**, as a representative example, revealed the lack of an NH_2_ absorption peak at 3292 and 3250 cm^−1^ and appearance of an absorption peak at 3149 cm^−1^ owing to the NH group. The ^1^H-NMR spectrum of **5c** exhibited new signals at δ 1.41, 7.33, and 7.37 ppm assigned to methyl and aromatic protons, in addition, the D_2_O exchangeable signal at δ 11.57 ppm due to cyclic NH. Its ^13^C-NMR spectrum of **5c** revealed the lack of C=S signal at 180 ppm and appearance 12 carbon signals. Moreover, the mass spectra of compounds **5a**–**c** gave molecular ion peaks at *m*/*z* 314, 328, and 348, respectively. This clearly indicates the carbothiohydrazide moiety was involved in cyclization reaction with hydrazonyl chlorides **2a**–**c** to give thiadiazine.

In a similar way, 6-(aryldiazenyl)-4*H*-1,3,4-thiadiazines **15a**–**c**, 6-(aryldiazenyl)-4*H*-1,3,4-thiadiazin-5(6*H*)-ones **17a**–**c**, and 5-aryl-4*H*-1,3,4-thiadiazines **20** were synthesized in very good yields from the reaction of pyrazole-1-carbothiohydrazide **1b** with hydrazonyl cholrides **2a**–**c**, **6a**–**c**, and α-haloketone **9**, respectively, under similar reaction condition as described before (Scheme 3). Compound **20** was previously synthesized from a one pot reaction of 3-(2-bromoacetyl)-2*H*-chromen-2-ones, thiocarbohydrazide, and pentane-2,4-dione [25]. The IR spectrum of **17b** revealed the lack of NH_2_ band present in the IR spectra of starting pyrazole **1b** and the appearance of new absorption bands at 3176 and 1680 cm^−1^ corresponding to NH and CO functional groups, respectively. Likewise, the ^1^H-NMR spectra showed a new singlet signal at δ 3.11 ppm due to H-6 of thiadiazine, two doublet signals at δ 7.13, 7.25 ppm integrated for four protons of 4-disubstitued benzene ring, and D_2_O-exchangeable signals at 11.23 ppm due to NH. Its ^13^C-NMR spectrum did not exhibit the C=S signal at 180 ppm which observed in the starting material, but instead displayed 13 carbon signals. The mass spectra of **17a**–**c** showed molecular ion peaks at *m*/*z* 314, 328, and 348, respectively, which were in an accord with the calculated masses (c.f. experimental section).

The coupling reaction of **1a** with 4-substituted arenediazonium chloride was performed in ethanol containing sodium acetate at 0–5 °C to give the corresponding hydrazones **21a**–**c**. Also, the reaction of **1a** with 4-(dimethylamino)benzaldehyde in ethanol containing few drops of HCl gave *N*′-(4-(dimethylamino)benzylidene)-3-methyl-5-oxo-4,5-dihydro-1*H*-pyrazole-1-carbothiohydrazide **22** (Scheme 4). The ^1^H-NMR spectra of **21a**–**c** showed lack the singlet signal due to CH_2_ (C-4 of pyrazolone) and showed an aromatic multiplets in the region 7.22–7.84 ppm. In addition, new D_2_O-exchangeable signals appeared in the region 11.74–12.24 ppm. These data support the successful coupling C-4 of pyrazolone with 4-substituted arenediazonium chloride. The IR spectrum of hydrazone **22** did not show an NH_2_ band. The ^1^H-NMR spectrum of **22** showed a new singlet signal at 9.65 ppm due to the azamethine proton (N=CH) and an aromatic multiplets at 6.80 and 7.91 ppm. Also, the mass spectra of **21a**–**c** and **22** are in an agreement with the calculated masses.

### 2.2. Antimicrobial Activity

In vitro antimicrobial screening of the newly synthesized compounds was carried out by the agar diffusion method using cultures of two fungal strains (*Candida albicans* (ATCC 10231) and *Aspergillus niger* (ATCC 16404), as well as four bacteria strains, two Gram positive bacteria (*Staphylococcus aureus* (ATCC 29213), *Bacillus subtilus* (ATCC 6051), and two Gram negative bacteria (*Klebsiella pneumoniae* (ATCC 700603) and *Escherichia coli* (ATCC 25922). The standard antibiotic Chloramphenicol and Antifungal Clotrimazole was used as controls to evaluate the potency of the compounds being studied under the same conditions.

As shown in Table 1 compounds **5b**, **8a**, **12**, and **17a** were found to be inactive against all microorganisms while compounds **5a**, **5c**, **8c**, **15a**, **15b**, **15c**, **17b**, **17c**, and **20** exhibited low activity against some microorganisms only and inactive against others. Compounds **8b** showed good activities against fungi and Gram positive bacteria. Compound **22** displayed good activities against all microorganisms except *Candida albicans* did not show any activity. Compounds **21a**–**c** displayed a broad spectrum activity against all microorganisms. Compound **21c** showed the highest activity against *Candida albicans* with inhibition zones of 25 mm while compound **21a** showed the highest activity against other strains, e.g., *Aspergillus niger*, *Staphylococcus aureus*, *Bacillus subtilus*, *Klebsiella pneumoniae*, and *Escherichia coli* with inhibition zones 35, 22, 30, 20, and 27 mm, respectively. The variation in the effectiveness of different compounds against microorganism depends on either the impermeability of the cells of the microbes or on differences in the ribosomes of microbial cells [26]. It may be concluded that the antimicrobial activity of the compounds is related to the cell wall structure of the bacterium as well as the structure of the pyrazole derivatives itself. It is possible because the cell wall is essential to the survival of bacteria and some antibiotics are able to kill bacteria by inhibiting a step in the synthesis of peptidoglycan. Gram-positive bacteria possess a thick cell wall containing many layers of peptidoglycan and teichoic acids, but in contrast, Gram negative bacteria have a relatively thin cell wall consisting of a few layers of peptidoglycan surround by a second lipid membrane containing lipopolysaccharides and lipoproteins. These differences in cell wall structure can produce differences in antibacterial susceptibility and some antibiotics can kill only Gram-positive bacteria and are ineffective against Gram-negative pathogens [27].

On the other hand, it is obvious that compounds **21a** > **21b** > **21c** exhibited potent inhibition activity owing to its characteristic skeleton that containing free carbothiohydrazide moiety that confer its softness, six donating N atoms, and planar 4-substituted phenyl group compared with the other pyrazolyl 1,3,4-thiadiazine derivatives.

The compounds which showed greater antibacterial and antifungal activities were further assayed for minimum inhibitory concentration (MIC), and the values are listed in Table 2. MIC is the lowest concentration of an antimicrobial that will inhibit the visible growth of a microorganism. Compounds **21a** displayed low MIC value on *Aspergillus niger*, *Staphylococcus aureus, B. subtilis*, and *Klebsiella pneumoniae* than standard drug Clotrimazole and Chloramphenicol and showed MIC value on *Candida albicans* and *Escherichia coli* equal to standard drugs. The MIC value of compound **21b** against *Aspergillus niger*, *B. subtilis*, and *Klebsiella pneumoniae* was equal to standard drugs Clotrimazole and Chloramphenicol. Moreover, Compound **21c** showed a MIC value on *Aspergillus niger Staphylococcus aureus, B. subtilis*, *Klebsiella pneumonia*, and *Escherichia coli* equal to the standard drugs. The structure–activity relationship revealed that compounds with pyrazole-1-carbothiohydrazide unit **21a**, **21b**, **21c**, and **22** showed higher activity than compounds have pyrazolyl thiadiazine unit. Further, the presence of free carbothiohydrazide moiety increases the activity of **21a**–**c** and the presence of electron donating substituents at the aromatic ring increased the activity of **21a**.

## 3. Experimental Section

### 3.1. General Information

All melting points were determined on a digital Gallen-Kamp MFB-595 instrument (Gallenkamp, London, UK) using open capillary tubes and are uncorrected. IR spectra were recorded on a Schimadzu FTIR 440 spectrometer (Shimadzu, Tokyo, Japan) using KBr pellets. Mass spectra were performed at 70 eV on an MS-50 Kratos (A.E.I.) spectrometer (Shimadzu, Tokyo, Japan) provided with a data system. ^1^H-NMR and ^13^C-NMR spectra were recorded on a Bruker model (500 MHz) Ultra Shield NMR spectrometer (Bruker, Coventry, UK) in CDCl_3_ or DMSO-d_6_ using tetramethylsilane (TMS) as an internal standard; chemical shifts are reported as δ ppm units. Solvents were dried by standard techniques. The monitoring of the progress of all reactions and homogeneity of the synthesized compounds was carried out and was run using thin layer chromatography (TLC) aluminum sheets silica gel 60 F_254_ (Merck, Darmstadt, Germany). Compound **1b** was prepared previously by Alekseev et al. [24].

### 3.2. Synthesis

*3-Methyl-5-oxo-4,5-dihydro-1H-pyrazole-1-carbothiohydrazide* (**1a**). Thiocarbohydrazide (10.6 g, 0.1 mol) was dissolved in a mixture of ethanol (20 mL) and HCl (1 mL) and ethyl acetoacetate was added (13 mL, 0.1 mol). The mixture was refluxed for 1 h. After cooling, the white precipitate was filtered off, washed with ethanol, and dried under reduced pressure. White crystals, yield (92%), m.p. 135–136 °C. IR (KBr) *ν* (cm^−1^): 3292, 3250, 3182 (NH_2_ & NH), 1685 (C=O), 1647 (C=N), ^1^H-NMR (500 MHz, CDCl_3_) δ (ppm): 2.02 (s, 3H, CH_3_), 3.28 (s, 2H, pyrazole-H_4_), 8.7 (d, D_2_O exchangeable, 2H, NH_2_), 10.18 (s, D_2_O exchangeable, 1H, NH); ^13^C-NMR (125 MHz, CDCl_3_) δ_C_ (ppm): 15.5 (CH_3_), 43.0 (CH_2_, pyrazole-C_4_), 160.5 (pyrazole-C_3_), 166 (C=O), 179 (C=S); MS *m*/*z* (%): 172 [M]^+^ (11%), 130 (100); Anal. Calcd. for C_5_H_8_N_4_OS (172.21): C, 34.87; H, 4.68; N, 32.54, Found: C, 34.57; H, 4.50; N, 32.41%.

#### 3.2.1. General procedure for synthesis compounds **5**, **8**, **12**, **15**, **17**, and **20**

Equimolar amounts of **1a** or **1b** (1 mmol) and 2-oxo-*N*-arylpropanehydrazonoyl chloride **2a**–**c**; ethyl 2-chloro-2-(2-arylhydrazineylidene)acetate **6a**–**c** or 3-(2-bromoacetyl)-2*H*-chromen-2-one **8** (1 mmol) in absolute ethanol (30 mL) (few drops of triethylamine was added in case of **2a**–**c** and **6a**–**c**) was heated under reflux for 3–6 h (TLC), then left to cool. The solid was isolated by filtration, washed with ethanol, dried, and recrystallized from (EtOH).

*5-Methyl-2-(5-methyl-6-(phenyldiazenyl)-4H-1,3,4-thiadiazin-2-yl)-2,4-dihydro-3H-pyrazol-3-one* (**5a**). Red crystals, yield (85%), m.p. 193–194 °C (EtOH); IR (*ν*_max_, cm^−1^): 3242 (NH), 1690 (C=O), 1600 (C=N), 1600–1440 (C=C); ^1^H-NMR (500 MHz, CDCl_3_) δ_H_ (ppm): 1.25 (s, 3H, CH_3_), 1.44 (s, 3H, CH_3_), 3.10 (s, 2H, pyrazole-H_4_), 7.33 (t, 2H, Ar-H), 7.44 (t, 2H, Ar-H), 8.04 (d, 1H, *J* = 8.5 Hz, Ar-H), 11.52 (s, D_2_O exchangeable, 1H, NH); ^13^C-NMR (125 MHz, CDCl_3_) δ_C_ (ppm): 12.2 (CH_3_), 16.0 (CH_3_), 44.0 (CH_2_, pyrazole-C_4_), 95.4, 121.7, 129.7, 148.2, 151.8, 154.6, 160.5, 166.3 (C=O); MS *m*/*z* (%): 314 [M]^+^ (21%), 245(100); Anal. Calcd. for C_14_H_14_N_6_OS (314.37): C, 53.49; H, 4.49; N, 26.73, Found: C, 53.69; H, 4.19; N, 26.67%.

*5-Methyl-2-(5-methyl-6-(p-tolyldiazenyl)-4H-1,3,4-thiadiazin-2-yl)-2,4-dihydro-3H-pyrazol-3-one* (**5b**). Brown powder, yield (86%), m.p. 219–220 °C (EtOH); IR (*ν*_max_, cm^−1^):, 3244 (NH), 1687 (C=O), 1591 (C=N), 1558–1440 (C=C); ^1^H-NMR (500 MHz, CDCl_3_) δ_H_ (ppm): 1.27 (s, 3H, CH_3_), 1.48 (s, 3H, CH_3_), 1.54 (s, 3H, CH_3_), 3.11 (s, 2H, pyrazole-H_4_), 7.37 (dd, 2H, *J* = 8.5, 2.5 Hz, Ar-H), 7.82 (dd, 2H, *J* = 7.6, 2.5 Hz, Ar-H), 11.54 (s, D_2_O exchangeable, 1H, NH); ^13^C-NMR (125 MHz, CDCl_3_) δ_C_ (ppm): 12.9 (CH_3_), 16.5 (CH_3_), 21.5 (CH_3_), 42.9 (CH_2_, pyrazole-C_4_), 94.9, 128.7, 129.9, 138.6, 146.1, 152.4, 155.4, 160.7, 165.7 (C=O); MS *m*/*z* (%): 328 [M]^+^ (31%), 245 (100); Anal. Calcd. for C_15_H_16_N_6_OS (328.39): C, 54.86; H, 4.91; N, 25.59, Found: C, 55.01; H, 4.67; N, 25.51%.

*2-(6-((4-Chlorophenyl)diazenyl)-5-methyl-4H-1,3,4-thiadiazin-2-yl)-5-methyl-2,4-dihydro-3H-pyrazol-3-one* (**5c**). Brown powder, yield (87%), m.p. 230–231 °C (EtOH); IR (*ν*_max_, cm^−1^): 3149 (NH), 1692 (C=O), 1654 (C=N), 1593–1462 (C=C); ^1^H-NMR (500 MHz, CDCl_3_) δ_H_ (ppm): 1.22 (s, 3H, CH_3_), 1.41 (s, 3H, CH_3_), 2.53 (s, 2H, pyrazole-H_4_), 7.33 (dd, 2H, *J* = 9, 2.5 Hz, Ar-H), 7.37 (dd, 2H, *J* = 7, 2.5 Hz, Ar-H), 11.57 (s, D_2_O exchangeable, 1H, NH); ^13^C-NMR (125 MHz, CDCl_3_) δ_C_ (ppm): 12.2 (CH_3_), 15.8 (CH_3_), 43.4 (CH_2_, pyrazole-C_4_), 96.0, 129.7, 131.7, 134.8, 147.0, 151.6, 154.0, 161.4, 165.6 (C=O); MS *m*/*z* (%): 348 [M]^+^ (35%), 350 [M + 2]^+^ (10), 245(100); Anal. Calcd. for C_14_H_13_ClN_6_OS (348.81): C, 48.21; H, 3.76; N, 24.09, Found: C, 48.62; H, 3.46; N, 24.14%.

*2-(3-Methyl-5-oxo-4,5-dihydro-1H-pyrazol-1-yl)-6-(phenyldiazenyl)-4H-1,3,4-thiadiazin-5(6H)-one* (**8a**). Yellow crystals, yield (87%), m.p. 220–221 °C (EtOH); IR (*ν*_max_, cm^−1^): 3180 (NH), 1695 (C=O), 1681 (C=O), 1633 (C=N), 1598–1496 (C=C); ^1^H-NMR (500 MHz, CDCl_3_) δ_H_ (ppm): 2.14 (s, 3H, CH_3_), 2.27 (s, 2H, pyrazole-H_4_), 3.52 (s, 1H, thiadiazine-H_6_), 7.31 (t, 2H, Ar-H), 7.45 (t, 2H, Ar-H), 7.64 (d, 1H, *J* = 8.5 Hz, Ar-H), 10.6 (s, D_2_O exchangeable, 1H, NH); ^13^C-NMR (125 MHz, CDCl_3_) δ_C_ (ppm): 14.9 (CH_3_), 42, 81.5, 121.4, 126.5, 129.5, 151.4, 152.9, 159.7, 165 (C=O), 169 (C=O); MS *m*/*z* (%): 316 [M]^+^ (20%), 350 (8), 98 (100); Anal. Calcd. for C_13_H_12_N_6_O_2_S (316.34): C, 49.36; H, 3.82; N, 26.57, Found: C, 49.06; H, 3.79; N, 26.35%.

*2-(3-Methyl-5-oxo-4,5-dihydro-1H-pyrazol-1-yl)-6-(p-tolyldiazenyl)-4H-1,3,4-thiadiazin-5(6H)-one* (**8b**). Yellow crystals, yield (84%), m.p. 238–239 °C (EtOH); IR (*ν*_max_, cm^−1^): 3176 (NH), 1699 (C=O), 1695 (C=O), 1621 (C=N), 1593–1506 (C=C); ^1^H-NMR (500 MHz, CDCl_3_) δ_H_ (ppm): 2.16 (s, 3H, CH_3_), 2.22 (s, 2H, pyrazole-H_4_), 3.42 (s, 1H, thiadiazine-H_6_), 3.45 (s, 3H, CH_3_), 7.2 (d, 2H, *J* = 8 Hz, Ar-H), 7.3 (dd, 2H, *J* = 6.5, 2 Hz, Ar-H), 10.9 (s, D_2_O exchangeable, 1H, NH); ^13^C-NMR (125 MHz, CDCl_3_) δ_C_ (ppm): 14.5 (CH_3_), 21 (CH_3_), 42 (CH_2_), 79.5, 123.4, 129.5, 138.4, 148, 151.4, 159.7, 165 (C=O), 169 (C=O); MS *m*/*z* (%): 330 [M]^+^ (25%), 350 (8), 98 (100); Anal. Calcd. for C_14_H_14_N_6_O_2_S (330.37): C, 50.90; H, 4.27; N, 25.44, Found: C, 50.43; H, 4.15; N, 25.27%.

*6-((4-Chlorophenyl)diazenyl)-2-(3-methyl-5-oxo-4,5-dihydro-1H-pyrazol-1-yl)-4H-1,3,4-thiadiazin-5(6H)-one* (**8c**). Yellow powder, yield (81%), m.p. 260–261 °C (EtOH); IR (*ν*_max_, cm^−1^): 3184 (NH), 1693, 1689 (C=O), 1625 (C=N), 1583–1529 (C=C); ^1^H-NMR (500 MHz, CDMSO-*d*_6_) δ_H_ (ppm): 2.08 (s, 3H, CH_3_), 2.13 (s, 2H, pyrazole-H_4_), 3.74 (s, 1H, thiadiazinone-H_6_), 7.24 (dd, 2H, *J* = 7, 2 Hz, Ar-H), 7.33 (dd, 2H, *J* = 9, 2 Hz, Ar-H), 10.58 (s, D_2_O exchangeable, 1H, NH); ^13^C-NMR (125 MHz, CDCl_3_) δ_C_ (ppm): 14.7 (CH_3_), 42 (CH_2_), 78, 123.9, 129.5, 134.4, 148, 153, 159.5, 164 (C=O), 168 (C=O); MS *m*/*z* (%): 349.87 [M]^+^ (2%), 293.77 (100); Anal. Calcd. for C_13_H_11_ClN_6_O_2_S (350.78): C, 44.51; H, 3.16; N, 23.96, Found: C, 44.19; H, 3.02; N, 23.62%.

*5-Methyl-2-(5-(2-oxo-2H-chromen-3-yl)-6H-1,3,4-thiadiazin-2-yl)-2,4-dihydro-3H-pyrazol-3-one* (**12**). Brown powder, yield (87%), m.p. 220–221 °C (EtOH); IR (*ν*_max_, cm^−1^): 1692, 1681 (2C=O), 1606 (C=N), 1558–1452 (C=C); ^1^H-NMR (500 MHz, CDCl_3_) δ_H_ (ppm): 2.17 (s, 3H, CH_3_), 3.42 (s, 2H, pyrazole-H_4_), 4.97 (s, 2H, thiadiazine-H_6_), 7.45 (dd, 1H, *J* = 7.5, 3.5Hz, coumarin-H_8_), 7.66 (dd, 1H, *J* = 6, 1.5 Hz, coumarin-H_6_), 7.81 (dd, 1H, *J* = 7.5, 1.5 Hz, coumarin-H_7_), 8.1 (dd, 1H, *J* = 7.8, 1, coumarin-H_5_), 8.94 (s, 1H, coumarin-H_4_); ^13^C-NMR (125 MHz, CDCl_3_) δ_C_ (ppm): 13.7 (CH_3_), 41.5 (CH_2_), 43.4 (CH_2_), K 116.4, 122.7, 128.6, 130.8, 135.2, 141.9, 144.5, 148.7, 152.7, 154.7, 158.5, 163.6, 190.3; MS *m*/*z* (%): 340 [M]^+^ (13%), 105 (100); Anal. Calcd. for C_16_H_12_N_4_O_3_S (340.36): C, 56.46; H, 3.55; N, 16.46, Found: C, 56.19; H, 3.29; N, 16.28.

*2-(3,5-Dimethyl-1H-pyrazol-1-yl)-5-methyl-6-(phenyldiazenyl)-4H-1,3,4-thiadiazine* (**15a**). Brown powder, yield (85%), m.p. 180–181 °C (EtOH); IR (*ν*_max_, cm^−1^): 3151 (NH), 1657 (C=N), 1598–1489 (C=C); ^1^H-NMR (500 MHz, CDCl_3_) δ_H_ (ppm): 2.25 (s, 3H, CH_3_), 2.29 (s, 3H, CH_3_), 2.57 (s, 3H, CH_3_), 6.05 (s, 1H, H4 pyrazole), 7.27 (t, 2H, Ar-H), 7.42 (t, 2H, Ar-H), 8.04 (d, 1H, *J* = 8.5 Hz, Ar-H), 11.54 (s, D_2_O exchangeable, 1H, NH); ^13^C-NMR (125 MHz, CDCl_3_) δ_C_ (ppm): 8.85 (CH_3_), 14.3 (CH_3_), 14.9 (CH_3_), 110, 117.5, 128, 129, 142.0, 148.5, 150.2, 155.0, 160, 189.5; MS *m*/*z* (%): 312 [M]^+^ (10%), 239.9 (100); Anal. Calcd. for C_15_H_16_N_6_S (312.40): C, 57.67; H, 5.16; N, 26.90, Found: C, 57.39; H, 5.04; N, 26.55%.

*2-(3,5-Dimethyl-1H-pyrazol-1-yl)-5-methyl-6-(p-tolyldiazenyl)-4H-1,3,4-thiadiazine* (**15b**). Brown powder, yield (86%), m.p. 205–206 °C (EtOH); IR (*ν*_max_, cm^−1^): 3156 (NH), 1683 (C=N), 1591–1456 (C=C); ^1^H-NMR (500 MHz, CDCl_3_) δ_H_ (ppm): 2.26 (s, 3H, CH_3_), 2.32 (s, 3H, CH_3_), 2.51 (s, 3H, CH_3_), 2.57 (s, 3H, CH_3_), 6.04 (s, 1H, pyrazole-H_4_), 7.32 (dd, 2H, *J* = 6 Hz, Ar-H), 7.86 (dd, 2H, *J* = 8.5 Hz, Ar-H), 11.52 (s, D_2_O exchangeable, 1H, NH); ^13^C-NMR (125 MHz, CDCl_3_) δ_C_ (ppm): 8.54 (CH_3_), 13.2 (CH_3_), 18.6 (CH_3_), 18.6 (CH_3_), 103.3, 114.9, 121.0, 129.1, 129.3, 129.6, 148.9, 150.6, 159.2, 189.5; MS *m*/*z* (%): 326 [M]^+^ (30%), 299 (100); Anal. Calcd. for C_16_H_18_N_6_S (326.42): C, 58.87; H, 5.56; N, 25.75, Found: C, 58.49; H, 5.27; N, 25.41%.

*6-((4-Chlorophenyl)diazenyl)-2-(3,5-dimethyl-1H-pyrazol-1-yl)-5-methyl-4H-1,3,4-thiadiazine* (**15c**). Brown crystals, yield (84%), m.p. 187–188 °C (EtOH); IR (*ν*_max_, cm^−1^): 3153 (NH), 1654 (C=N), 1595–1487 (C=C); ^1^H-NMR (500 MHz, CDCl_3_) δ_H_ (ppm): 2.25 (s, 3H, CH_3_), 2.29 (s, 3H, CH_3_), 2.57 (s, 3H, CH_3_), 6.05 (s, 1H, pyrazole-H_4_), 7.33 (dd, 2H, *J* = 9, Ar-H), 7.98 (dd, 2H, *J* = 9, Ar-H), 11.56 (s, D_2_O exchangeable, 1H, NH); ^13^C-NMR (125 MHz, CDCl_3_) δ_C_ (ppm): 8.58 (CH_3_), 13.3 (CH_3_), 14.4 (CH_3_), 111, 116.4, 126.8, 128.9, 141.0, 149.6, 151.2, 154.0, 159.2, 189.5; MS *m*/*z* (%): 348.97 [M + 3]^+^ (20%), 345.94 [M]^+^ (10%), 317 (100); Anal. Calcd. for C_15_H_15_ClN_6_S (346.84): C, 51.95; H, 4.36; N, 24.23, Found: C, 51.60; H, 4.15; N, 24.07%.

*2-(3,5-dimethyl-1H-pyrazol-1-yl)-6-(phenyldiazenyl)-4H-1,3,4-thiadiazin-5(6H)-one* (**17a**). Green powder, yield (85%), m.p. 126–127 °C (EtOH); IR (*ν*_max_, cm^−1^): 3178 (NH), 1681 (C=O), 1602 (C=N), 1575–1473 (C=C); ^1^H-NMR (500 MHz, CDCl_3_) δ_H_ (ppm): 2.12 (s, 3H, CH_3_), 2.24 (s, 3H, CH_3_), 3.2 (s, 1H, H6 thiadiazinone), 6 (s, 1H, pyrazole-H_4_), 7.33 (t, 2H, Ar-H), 7.44 (t, 2H, Ar-H), 7.65 (d, 1H, *J* = 8.5 Hz, Ar-H), 11 (s, D_2_O exchangeable, 1H, NH); ^13^C-NMR (125 MHz, CDCl_3_) δ_C_ (ppm): 12.7 (CH_3_), 14.9 (CH_3_), 85.2, 114, 121.4, 129.5, 135.4, 142.9, 145.7, 148.6, 152.0, 169.1; MS *m*/*z* (%):314 [M]^+^ (45), 350 (8), 86 (100); Anal. Calcd. for C_14_H_14_N_6_OS (314.37): C, 53.49; H, 4.49; N, 26.73, Found: C, 53.17; H, 4.19; N, 26.43%.

*2-(3,5-Dimethyl-1H-pyrazol-1-yl)-6-(p-tolyldiazenyl)-4H-1,3,4-thiadiazin-5(6H)-one* (**17b**). Green powder, yield (80%), m.p. 164–165 °C (EtOH); IR (*ν*_max_, cm^−1^): 3176 (NH), 1680 (C=O), 1612 (C=N), 1599–1465 (C=C); ^1^H-NMR (500 MHz, CDCl_3_) δ_H_ (ppm): 2.13 (s, 3H, CH_3_), 2.23 (s, 3H, CH_3_), 2.46 (s, 3H, CH_3_), 3.11 (s, 1H, thiadiazine-H_6_), 6.04 (s, 1H, pyrazole-H_4_), 7.13 (d, 2H, *J* = 8 Hz, Ar-H), 7.25 (dd, 2H, *J* = 6.5, 2 Hz, Ar-H), 11.23 (s, D_2_O exchangeable, 1H, NH); ^13^C-NMR (125 MHz, CDCl_3_) δ_C_ (ppm): 12.4 (CH_3_), 14.8 (CH_3_), 21.7 (CH_3_), 86.0, 113, 125.7, 130.7, 134.8, 144.0, 147.6, 149, 154.0, 169.6; MS *m*/*z* (%): 328 [M]^+^ (3%), 321(20), 148.8 (90), 85.9 (100); Anal. Calcd. for C_15_H_16_N_6_OS (328.39): C, 54.86; H, 4.91; N, 25.59, Found: C, 54.39; H, 4.69; N, 25.41%.

*6-((4-Chlorophenyl)diazenyl)-2-(3,5-dimethyl-1H-pyrazol-1-yl)-4H-1,3,4-thiadiazin-5(6H)-one* (**17c**). Brown powder, yield 83%, m.p. 140–141 °C (EtOH); IR (*ν*_max_, cm^−1^): 3169 (NH), 1681 (C=O), 1614 (C=N), 1599–1541 (C=C); ^1^H-NMR (500 MHz, CDCl_3_) δ_H_ (ppm): 2.12 (s, 3H, CH_3_), 2.24 (s, 3H, CH_3_), 3.2 (s, 1H, H6 thiadiazinone), 6.05 (s, 1H, H4 pyrazole), 7.17 (d, 2H, *J* = 8 Hz, Ar-H), 7.30 (dd, 2H, *J* = 6.5, 2 Hz, Ar-H), 11.17 (s, D_2_O exchangeable, 1H, NH); ^13^C-NMR (125 MHz, CDCl_3_) δ_C_ (ppm): 12.4 (CH_3_), 14.8 (CH_3_), 81.0, 112, 124.2, 131.5, 135.6, 143.0, 146.7, 148.9, 153.0, 168.6; MS *m*/*z* (%): 348 [M]^+^ (25%), 350 (8), 86 (100); Anal. Calcd. for C_14_H_13_ClN_6_OS (348.81): C, 48.21; H, 3.76; N, 24.09, Found: C, 47.99; H, 3.62; N, 23.89%.

#### 3.2.2. General procedure for synthesis **21a**–**c**

To a stirred solution of compound **1a** (0.516 g, 3 mmol) in ethanol (30 mL) sodium acetate trihydrate (0.39 g, 3 mmol) was added. After stirring for 15 min, the mixture was chilled at 0 °C and treated with a cold solution of the respective aniline (4-chloroaniline (0.381 g, 3 mmol), *p*-toluidine (0.310 g, 3 mmol), or 4-aminobenzenesulfonamide (0.516 g, 3 mmol)) in 6 M hydrochloric acid (1.5 mL) with a sodium nitrite solution (0.21 g, 3 mmol, in 3 mL water). The addition of the diazonium salt was stirred for an additional 2 h at 0–5 °C and then left for 8 h in a refrigerator (4 °C). The resulting solid was collected by filtration, washed thoroughly with water, and dried. The crude product was crystallized from ethanol to give hydrazones **21a**–**c**.

*3-Methyl-5-oxo-4-(2-(p-tolyl)hydrazineylidene)-4,5-dihydro-1H-pyrazole-1-carbothiohydrazide* (**21a**). Brown powder, yield (80%), m.p. 120–121 °C (EtOH); IR (*ν*_max_, cm^−1^): 3254–3142 (NH_2_ & 2NH), 1698 (C=O), 1595 (C=N), 1556–1485 (C=C); ^1^H-NMR (500 MHz, CDMSO-*d*_6_) δ_H_ (ppm): 2.31 (s, 3H, CH_3_), 2.35 (s, 3H, CH_3_), 6.85 (d, D_2_O exchangeable, 2H, NH_2_), 7.46 (m, 4H, Ar-H), 11.31 (s, D_2_O exchangeable, 1H, NH), 11.74 (s, D_2_O exchangeable, 1H, NH); ^13^C-NMR (125 MHz, DMSO-*d*_6_) δ_C_ (ppm): 11.9 (CH_3_), 21.4 (CH_3_), 116.4, 127.4, 128.4, 130, 142.1, 147.5, 163.8, 194.0; MS *m*/*z* (%): 290 [M]^+^ (5%), 95 (100); Anal. Calcd. for C_12_H_14_N_6_OS (290.09): C, 49.64; H, 4.86; N, 28.95, Found: C, 49.41; H, 4.64; N, 28.77%.

*4-(2-(4-Chlorophenyl)hydrazineylidene)-3-methyl-5-oxo-4,5-dihydro-1H-pyrazole-1-carbothiohydrazide* (**21b**). Red crystals, yield (85%), m.p. 117–118 °C (EtOH); IR (*ν*_max_, cm^−1^): 3383–3172 (NH_2_ & 2NH), 1674 (C=O), 1593 (C=N), 1544–1481 (C=C); ^1^H-NMR (500 MHz, CDMSO-*d*_6_) δ_H_ (ppm): 2.31 (s, 3H, CH_3_), 6.99 (s, D_2_O exchangeable, 2H, NH_2_), 7.1 (s, D_2_O exchangeable, 1H, NH), 7.48 (m, 4H, Ar-H), 11.76 (s, D_2_O exchangeable, 1H, NH); ^13^C-NMR (125 MHz, DMSO-*d*_6_) δ_C_ (ppm): 11.9 (CH_3_), 116.4, 117.4, 118.4, 128, 129.5, 147.1, 163.8 (C=O), 194 (C=S); MS *m*/*z* (%): 310 [M]^+^ (5%), 285.78 (30), 267.95 (90); Anal. Calcd. for C_11_H_11_ClN_6_OS (310.76): C, 42.52; H, 3.57; N, 27.04, Found: C, 42.36; H, 3.29; N, 26.88%.

*4-(2-(1-(Hydrazinecarbonothioyl)-3-methyl-5-oxo-1,5-dihydro-4H-pyrazol-4-ylidene)hydrazineyl)benzenesulfonamide* (**21c**). Brown powder, yield (80%), m.p. 140–141 °C (EtOH); IR (*ν*_max_, cm^−1^): 3296–3221 (2NH_2_ & 2NH), 1693 (C=O), 1595 (C=N), 1543–1489 (C=C); ^1^H-NMR (500 MHz, CDMSO-*d*_6_) δ_H_ (ppm): 2.28 (s, 3H, CH_3_), 6.98 (s, D_2_O exchangeable, 2H, NH_2_), 7.08 (s, D_2_O exchangeable, 2H, NH_2_), 7.18 (s, D_2_O exchangeable, 2H, NH_2_), 7.72 (d, 2H, *J* = 7.5 Hz, Ar-H), 7.84 (d, 2H, *J* = 8.5 Hz, Ar-H), 11.31 (s, D_2_O exchangeable, 1H, NH), 12.24 (s, D_2_O exchangeable, 1H, NH); ^13^C-NMR (125 MHz, DMSO-*d*_6_) δ_C_ (ppm): 11.8 (CH_3_), 116.6, 126.8, 140.9, 144, 153.8, 160.7, 169 (C =O), 180 (C=S); MS *m*/*z* (%): 354.93 [M]^+^ (4%), 255.68 (100); Anal. Calcd. for C_11_H_13_N_7_O_3_S_2_ (355.39): C, 37.18; H, 3.69; N, 27.59, Found: C, 36.98; H, 3.56; N, 27.47%.

*Synthesis of N′-(4-(Dimethylamino)benzylidene)-3-methyl-5-oxo-4,5-dihydro-1H-pyrazole-1-carbothiohydrazide* (**22**). A few drops of HCl were added to a mixture of **1a** (0.516 g, 3 mmol), and 4-(dimethylamino)benzaldehyde (0.447 g, 3 mmol) in EtOH (20 mL), and the reaction mixture was stirred 6 h. The precipitate formed was collected by filtration, dried, washed with EtOH, and recrystallized from EtOH. Red powder, yield (89%), m.p. 261–262 °C (EtOH); IR (*ν*_max_, cm^−1^): 3269 (NH), 1689 (C=O), 1510 (C=N), 1504–1456 (C=C); ^1^H-NMR (500 MHz, CDMSO-*d*_6_) δ_H_ (ppm): 2.68 (s, 3H, CH_3_), 3 (s, 6H, N(CH_3_)_2_), 3.04 (s, 2H, CH_2_, pyrazole-4), 6.80 (m, 2H, Ar-H), 7.91 (m, 2H, Ar-H), 9.65 (s, 1H, CH=N), 11.97 (s, D_2_O exchangeable, 1H, NH); ^13^C-NMR (125 MHz, DMSO-*d*_6_) δ_C_ (ppm): 16.6 (CH_3_), 40 (CH_3_), 41 (CH_2_),111.6, 124, 129.5, 131.3, 154.4, 158, 163 (C=O), 190.5 (C=S); MS *m*/*z* (%): 290 [M]^+^ (25%), Anal. Calcd. for C_14_H_17_N_5_OS (303.38): C, 55.43; H, 5.65; N, 23.08, Found: C, 55.13; H, 5.30; N, 22.97%.

### 3.3. Antimicrobial Evaluation

The antibacterial activity of the synthesized compounds was tested against a panel of two gram positive bacteria (*Staphylococcus aureus* and *Bacillus subtilis*) and two Gram-negative bacteria (*Klebsiella pneumoniae* and *Escherichia coli*). The antifungal activities of the compounds were tested against two fungi (*Candida albicans* and *Aspergillus flavus*). A solution of each compounds in DMSO with concentration 1 mg/mL was prepared separately, paper discs of Whatman filter paper were prepared with standard size (5 cm) were cut and sterilized in an autoclave. The paper discs soaked in the desired concentration of the compound solution were placed aseptically in the petri dishes containing nutrient agar media (agar 20 g + beef extract 3 g + peptone 5 g) seeded with *Staphylococcus aureus*, *Bacillus subtilis*, *E. coli*, *Pseudomonas aeuroginosa*, *Candida albicans*, and *Aspergillus flavus*. The petri dishes were incubated at 36 °C and the inhibition zones were recorded after 24 h of incubation. Each treatment was replicated three times. The antibacterial activity of a common standard antibiotic chloramphenicol and antifungal clotrimazole were also recorded using the same procedure as above at the same concentration and solvents [28].

The MIC was determined using the disc diffusion technique by preparing discs containing 1.9–1000 µg/mL of each compound against gram positive, gram negative, and fungi. Twofold dilutions of the solution were prepared. The microorganism suspensions at 10 CFU/mL (colony forming unit/mL) concentrations were inoculated to the corresponding wells. The plates were incubated at 36 °C for 24 h for the bacteria. The standard antibiotic chloramphenicol and antifungal clotrimazole was also recorded using the same procedure as above at the same concentration and solvents. At the end of the incubation period, the minimum inhibitory concentrations (MIC) values were recorded as the lowest concentration of the substance that had no visible turbidity. Control experiments with DMSO and uninoculated media were run parallel to the test compounds under the same condition [28].

## 4. Conclusions

A novel series of pyrazole and pyrazolone derivatives was synthesized, in good yields, starting from pyrazole-1-carbothiohydrazide **1a**,**b**. A number of prepared compounds showed moderate to good antimicrobial activities. Hydrazones **21a**–**c** showed significant antimicrobial activities with MIC values equal to or lower than standard drugs chloramphenicol and clotrimazole. It is clearly that the presence of free carbothiohydrazide moiety increases antimicrobial activity. 

## Figures and Tables

**Table 1:** In vitro antimicrobial activity of the synthesized compounds ^a,b^.

Comp. No.	Fungi		Gram Positive Bacteria		Gram Negative Bacteria	
	*Candida albicans*	*Aspergillus niger*	*Staphylococcus aureus*	*Bacillus subtilus*	*Klebsiella pneumoniae*	*Escherichia coli*
**5a**	15 ± 1	20 ± 1.53	na ^c^	na	na	17 ± 1.00
**5b**	na	na	na	na	na	na
**5c**	12 ± 1.53	na	na	na	na	11 ± 0.58
**8a**	na	na	na	na	na	na
**8b**	20 ± 1.53	15 ± 2.65	13 ± 0.58	18 ± 0.58	11 ± 0.58	na
**8c**	9 ± 0.58	na	na	15 ± 2.08	na	na
**12**	na	na	na	na	na	na
**15a**	na	12 ± 0.00	na	na	na	na
**15b**	na	12 ± 0.58	na	na	na	na
**15c**	na	na	15 ± 0.58	29 ± 1.00	15 ± 1.15	23 ± 1.53
**17a**	na	na	na	na	na	na
**17b**	na	na	na	15 ± 0.58	na	14 ± 1.00
**17c**	na	na	17 ± 1.15	16 ± 0.58	na	16 ± 0.58
**20**	na	na	na	na	na	12 ± 0.58
**21a**	13 ± 0.00	35 ± 3.00	22 ± 1.15	30 ± 1.53	20 ± 0.58	27 ± 1.15
**21b**	18 ± 3.06	32 ± 0.58	21 ± 0.58	25 ± 0.58	18 ± 2.08	25 ± 0.58
**21c**	25 ± 3.00	29 ± 1.00	12 ± 1.53	23 ± 1.73	13 ± 0.58	25 ± 0.58
**22**	na	26 ± 0.58	15 ± 0.00	20 ± 0.58	17 ± 0.58	20 ± 1.53
Chloramphenicol	-	-	25 ± 0.58	30 ± 1.73	24 ± 1.15	24 ± 1.00
Clotrimazole	24 ± 4.51	20 ± 0.58	-	-	-	-

^a^ Antimicrobial activity expressed as inhibition diameter zones in millimeters (mm) of synthesized compounds against the pathological strains based on well diffusion assay. ^b^ The experiment was carried out in triplicate and the average zone of inhibition was calculated. ^c^ na No activity.

**Table 2:** Minimum inhibitory concentration (MIC) in (µg/mL) for compounds **8b**, **21a**–**c**, and **22**.

Minimum Inhibitory Concentration (MIC)						
Comp. No.	*Candida albicans*	*Aspergillus niger*	*Staphylococcus aureus*	*Bacillus subtilus*	*Klebsiella pneumoniae*	*Escherichia coli*
**8b**	125 ± 2.52	187.5 ± 0.50	375 ± 3.00	500 ± 3.51	500 ± 4.51	na
**21a**	7.8 ± 0.17	2.9 ± 0.06	125 ± 0.58	62.5 ± 0.50	62.5 ± 2.00	125 ± 2.52
**21b**	15.6 ± 0.76	5.8 ± 0.26	250 ± 8.08	125 ± 0.00	125 ± 2.65	187.5 ± 8.23
**21c**	11.6 ± 0.30	5.8 ± 0.65	187.5 ± 8.23	125 ± 1.00	125 ± 1.53	125 ± 0.00
**22**	93.7 ± 0.95	46.4 ± 0.84	250 ± 4.36	250 ± 4.58	250 ± 3.21	500 ± 8.00
Chloramphenicol	---	---	187.5 ± 0.06	125 ± 0.58	125 ± 3.51	125 ± 1.73
Clotrimazole	7.8 ± 0.06	5.8 ± 0.06	----	----	----	---

na: No activity.
